# The State of Public Health in Somalia: Top 5 Challenges and Strategies for Improvement

**DOI:** 10.1002/puh2.70138

**Published:** 2025-10-09

**Authors:** Yusuf Hared Abdi, Mohamed Sharif Abdi, Sharmake Gaiye Bashir, Naima Ibrahim Ahmed, Yakub Burhan Abdullahi

**Affiliations:** ^1^ Department of Environmental Science Faculty of Geoscience & Environment Hormuud University Mogadishu Somalia; ^2^ Demartino Public Hospital Mogadishu Somalia

## Abstract

As we navigate the evolving public health landscape in Somalia, this study provides a comprehensive analysis of critical challenges, including water, sanitation, and hygiene (WASH); malnutrition and food insecurity; infectious diseases; maternal and child health; and the rising burden of noncommunicable diseases (NCDs). The aftermath of prolonged conflict, economic instability, and environmental crises underscores the urgent need to strengthen healthcare infrastructure, enhance disease surveillance, and improve multisectoral collaboration. The complexities of governance, humanitarian aid, and healthcare access highlight the necessity of coordinated efforts between government institutions, NGOs, and international partners. Given the urgent need to combat infectious disease outbreaks and the persistent challenges of food insecurity and high maternal mortality rates, this study explores multifaceted issues, advocating targeted interventions, sustainable policies, and equitable healthcare delivery. Addressing the root causes of public health disparities requires commitment to resilience‐building, community engagement, and policy‐driven solutions. In this critical period, Somalia must embrace a unified vision that fosters collaboration, policy reform, and strategic investments, ensuring that public health remains a central pillar of national development and the humanitarian response.

## Graphical abstract

Somalia's public health system is severely strained by five interconnected challenges: inadequate WASH infrastructure, widespread malnutrition and food insecurity, high burden of infectious diseases, critical gaps in maternal and child health, and the rising prevalence of noncommunicable diseases (NCDs). These issues are compounded by fragile health governance, conflict, and environmental shocks. This review highlights the systemic nature of these challenges and presents integrated, multisectoral strategies centered on disease surveillance, health infrastructure, community engagement, and sustainable policy reform to build resilience and ensure equitable health service delivery across Somalia.

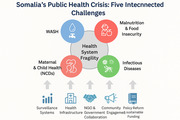

## 1 Introduction

Public health in Somalia faces significant challenges owing to a combination of factors, including the legacy of conflict, limited resources, and fragile infrastructure [1, 2]. The country's health infrastructure has been severely damaged by the civil war, resulting in a lack of essential resources, medical equipment, and trained personnel [1, 2]. Somalia's public health system is primarily supported by nongovernmental organizations (NGOs) and international agencies, as there is limited government capacity to deliver healthcare at the national level [2]. This dependency can lead to inconsistent service delivery and a lack of long‐term sustainability [2]. Additionally, access to healthcare remains a major challenge, particularly in rural and conflict‐affected areas, owing to geographic barriers, insecurity, and financial constraints [2]. The public health sector in Somalia is characterized by a shortage of appropriate guidelines, essential drugs, and diagnostic tools [3]. Infectious diseases, such as malaria, cholera, and measles, continue to be major public health threats, exacerbated by weak disease surveillance systems and inadequate access to clean water and sanitation [2]. Maternal and child health indicators are among the worst globally, with high mortality rates attributed to limited access to healthcare and various cultural and socioeconomic factors [2]. The main risk factors for mortality in Somalia include malnutrition, unsafe water, sanitation, and hand washing [4]. These factors contribute to nutritional deficiencies and enteric, neurological, musculoskeletal, and chronic respiratory diseases [4].

The challenges facing Somalia's public health system mirror the broader patterns observed across fragile states in sub‐Saharan Africa. Research from Nigeria, a country with similarly complex health system challenges, reveals how mistrust of health systems fundamentally undermines service utilization and health outcomes. Ogueji et al.’s [4] qualitative study of 54 health system professionals and community members identified systematic mistrust of health infrastructure, pharmaceutical quality, and provider competence as major barriers to healthcare access. Participants reported mistrust linked to dilapidated medical equipment, misdiagnosis due to poor laboratory services, shortage of qualified health workers, and absence of patient‐centered care [4]. These findings resonate strongly with Somalia's context, where similar infrastructure deficits, human resource shortages, and quality concerns create comparable barriers to health‐service utilization. The Nigerian study's recommendations, including government surveillance efforts, infrastructural development, computerization of healthcare operations, and investment in health worker training, provide valuable lessons for Somalia's health system strengthening efforts, particularly given the shared challenges of postconflict recovery and weak governance.

This study highlights five key public health challenges in Somalia: (1) water, sanitation, and hygiene; (2) malnutrition and food insecurity; (3) infectious diseases; (4) maternal and child health; and (5) noncommunicable diseases. These challenges were selected based on their profound impact on health outcomes, urgency, and opportunity for impactful public health interventions.

By examining these pressing issues, this study provides an in‐depth analysis of their implications for Somalia's public health landscape and explores potential strategies to address them. The aim is to offer insights that will empower policymakers, health practitioners, and researchers to implement targeted responses, strengthen public health systems, and improve the health and resilience of communities across the nation (Table 1).

**TABLE 1:** Major public health issues in Somalia and their underlying factors.

Public health challenge	Rationale for inclusion
Water, sanitation, and hygiene (WASH)	Inadequate WASH systems contribute to the spread of waterborne diseases, poor hygiene practices, and a lack of access to clean water, leading to severe health issues.
Malnutrition and food insecurity	Persistent droughts, conflict, and economic instability have led to food shortages, malnutrition, and stunting in vulnerable populations, particularly children.
Infectious diseases	Diseases like cholera, malaria, and tuberculosis remain significant public health threats, worsened by limited healthcare access and weak disease surveillance.
Maternal and child health	High maternal and child mortality rates result from inadequate healthcare services, poor nutrition, and a lack of skilled birth attendants.
Noncommunicable diseases (NCDs)	Chronic diseases such as diabetes, cardiovascular diseases, and cancer are increasing due to lifestyle changes, poor nutrition, and limited healthcare access.

## 2 Water, Sanitation, and Hygiene

Water, sanitation, and hygiene (WASH) challenges in Somalia are significant, impacting public health, especially access to clean and safe drinking water in rural and internally displaced person (IDP) communities [5]. Waterborne diseases, including cholera, diarrhea, and acute watery diarrhea (AWD), are highly prevalent in Somalia and are primarily caused by contaminated water sources [6, 7]. Between 2006 and 2015, Somalia reported the highest number of cholera cases in Southern and Eastern Africa [8]. The use of contaminated water greatly contributes to these health risks [9].

In addition, Somalia faces considerable challenges related to sanitation, including limited access to facilities, widespread open defecation, and inadequate waste management in both urban and rural areas [6]. Less than one in four people in Somalia had access to improved sanitation [6]. In rural villages, toilets and latrines are rare, with open defecation rates among the highest in the world [6]. A study using data from the Somali Health and Demographic Survey (SHDS) 2020 found that only 41.1% of households had improved latrines [10]. There are also significant hygiene barriers in Somalia, including low awareness and limited access to essential hygiene products, such as soap and menstrual hygiene supplies [5].

To improve access to safe water, expanding water supply systems is essential, which includes constructing and rehabilitating wells and boreholes as well as implementing desalination plants in coastal areas [7, 11]. Additionally, investing in effective waste management systems and promoting the construction of improved sanitation facilities are critical steps [11]. Moreover, improving sanitation situations by implementing public health campaigns is crucial to increasing hygiene awareness, promoting handwashing, and improving menstrual hygiene management practices, which can significantly reduce disease transmission [11, 12]. Strengthening mechanisms for responding to cholera and AWD, including early detection and treatment, is also crucial [6]. UNICEF has reached 892,566 people through hygiene promotion and the distribution of hygiene kits [11]. Finally, enhancing WASH governance and increasing funding at both the national and local levels are necessary for sustainable improvements [7]. This involves developing clear policies, ensuring accountability, and coordinating efforts among various governmental institutions [7].

## 3 Malnutrition and Food Insecurity

Somalia faces a significant challenge with a high prevalence of malnutrition, especially among children under the age of five [13, 14]. This is exacerbated by factors that contribute to food insecurity, including droughts, conflicts, and instability [15].

Malnutrition rates among children under five years of age in Somalia are alarmingly high, with projections indicating that nearly two in five children may suffer from acute malnutrition [16]. In 2023, 1.8 million children were affected [17]. This level of food insecurity significantly impacts public health and hinders economic stability [18]. Recurring droughts have devastating effects on food production and availability, leading to increased malnutrition in vulnerable populations [18]. For example, the failure of rains in 2021–2023 has led to displacement and increased the risk of morbidity and mortality [18]. This situation forces many people to choose between healthcare and food [18]. The crisis forced many families to choose between healthcare and food, with devastating consequences for individuals requiring long‐term treatment [14, 18].

International aid plays a crucial role in addressing immediate food needs [19, 20]. The European Union (EU), for example, supports eight humanitarian organizations, including Concern, in delivering emergency life‐saving interventions to communities affected by drought and conflict in Somalia [21]. However, long‐term solutions must focus on strengthening local food production, which faces numerous challenges, including climate‐related issues, underdeveloped markets, and limited access to high‐quality agricultural inputs [15, 22].

To combat malnutrition and enhance food security, Somalia must implement key policy recommendations, including strengthening local food production by promoting climate‐resilient agriculture, introducing drought‐resistant crops, and improving the irrigation infrastructure [20]. Targeted nutritional interventions should be developed to address seasonal food shortages and the impact of conflict on food access [23].

## 4 Infectious Diseases

The burden of infectious diseases remains a major public health challenge in Somalia and is exacerbated by fragile health systems and humanitarian crises [24]. Frequent disease outbreaks, poor access to healthcare, and limited vaccination coverage have contributed to the high morbidity and mortality rates, particularly among vulnerable populations [25]. Addressing these challenges requires a comprehensive approach that includes improved disease surveillance, strengthened healthcare infrastructure, and enhanced international collaboration [26].

Somalia faces a complex infectious disease burden characterized by endemic diseases (malaria and TB), epidemic‐prone diseases (cholera and measles), and emerging threats (COVID‐19 and antimicrobial resistance). The epidemiological pattern revealed distinct geographic clustering, with cholera predominantly affecting riverine areas and IDP settlements, while TB burden correlated with overcrowded urban centers and areas with high malnutrition rates [27, 28]. This spatiotemporal distribution reflects underlying vulnerabilities, including compromised immunity due to malnutrition, inadequate WASH infrastructure, and population mobility patterns [25].

Cholera outbreaks, often linked to inadequate water, sanitation, and hygiene (WASH) infrastructure, pose significant public health risks [29]. In early 2024, more than 10,000 cases and 120 deaths were reported across the country [24]. Contributing factors include the consumption of unsafe water, poor sanitation conditions, and open defecation, which facilitate contamination of food and water sources [29, 30].

Tuberculosis remains a persistent challenge, with Somalia ranking among the top 10 countries with a high burden of multidrug‐resistant TB [26]. In 2023, there were approximately 246 TB cases per 100,000 individuals [24]. Overcrowded and poorly ventilated living conditions, undernutrition, and limited health awareness further drive the transmission rates [24, 31]. Efforts to curb TB include strengthening vaccination programs, enhancing risk communication, and conducting research on vaccine hesitancy [32, 33].

Furthermore, NTDs are particularly prevalent in Somalia because of limited access to clean water, sanitation, and healthcare [34]. The most prevalent NTDs in Somalia are leprosy, schistosomiasis, soil‐transmitted helminthiases, and visceral leishmaniasis [34]. Approximately 5–6 million people in Somalia live in areas highly endemic for NTDs [34]. Somalia was classified as a global priority country for leprosy control in 2019 by the WHO Global Leprosy Programme, as it was one of the 16 countries reporting over 1000 new cases [34]. The World Health Organization (WHO) supports the Ministry of Health by providing technical support, mobilizing resources, and increasing coordination between donors and the Federal Government [34].

Despite ongoing efforts, Somalia faces significant obstacles in the control of infectious diseases [35]. Humanitarian crises, including mass displacement, malnutrition, and food insecurity, increase population vulnerability [25, 36]. Fragile health systems, weakened by prolonged conflict and political instability, hinder effective disease prevention and control [37]. Climate‐related shocks, such as droughts and floods, exacerbate these vulnerabilities and increase the risk of outbreaks [25]. Inadequate sanitation and hygiene practices, along with traditional beliefs, influence public perception of diseases and treatment‐seeking behavior [38]. Low vaccination rates contribute to recurrent outbreaks of preventable diseases, including measles and polio [39]. Furthermore, Somalia's heavy reliance on international aid complicates the sustainability of its health interventions, thereby making long‐term planning difficult [40].

To strengthen Somalia's health security, a multisectoral approach is essential. Improving disease surveillance through early warning systems can enable the rapid detection and response to outbreaks [25]. Address the identified diagnostic gaps through a tiered laboratory network approach: establishing regional reference laboratories for complex diagnostics (TB culture and antimicrobial sensitivity testing), strengthening district‐level capacity for rapid diagnostic tests (malaria RDTs and cholera rapid tests), and implementing mobile diagnostic units for hard‐to‐reach populations [25, 28]. This directly addresses the current 40% diagnostic gap identified in rural areas while building a sustainable local capacity. Raising public awareness of infectious diseases and the importance of immunization is a key strategy in combating misinformation and increasing vaccine uptake [41]. Community engagement plays a crucial role in infectious disease development [42]. Local strategies, such as tailored vaccination campaigns, can foster trust and combat misinformation [25, 42].

## 5 Maternal and Child Health

Somalia faces significant challenges in maternal and child health, marked by high mortality rates and various barriers to access care [43]. Somalia's maternal mortality trajectory reveals both challenges and progress. The maternal mortality ratio decreased from 1044 to 692 per 100,000 live births between 2006 and 2020, representing a 44% reduction [44]. This epidemiological profile indicates that targeted interventions addressing both emergency obstetric care and preventive services can reduce mortality. Child mortality rates are also distressingly high, with 68 infant deaths and 28 stillbirths per 1000 births, approximately double the average infant mortality rate in low‐ and middle‐income countries (LMICs) [43]. Somalia is among the 15 countries that the WHO has marked as very high‐alert countries for maternal, newborn, and under‐5 deaths [45].

Only 23% of the Somali mothers received maternal healthcare services [43]. Limited access to family planning services, with only 8% utilizing contraception and less than 1% using modern methods, contributes to unintended pregnancies [43]. Only 2.4% of mothers in Somalia received all forms of maternal healthcare services, in comparison to sub‐Saharan Africa (13.9%) and in South Asia (24.5%) [43].

Challenges exist at the community and health facility levels, including limited education, absence of sexual and reproductive health education, cultural factors such as early marriage, poor healthcare infrastructure, and ongoing conflict that disrupts healthcare delivery [43, 46]. Geographic disparities exacerbate health access issues, with healthcare workers concentrated in urban areas, leaving rural communities underserved [45]. Only 9.7% of the women attended four or more antenatal care (ANC4+) visits [45]. Skilled providers received less than 14% of the deliveries. Only 2.7% of the women had a postnatal care (PNC) check within 48 h after delivery [45]. Low levels of education contribute to a lack of awareness of family planning, while stigma surrounding reproductive health perpetuates misconceptions about contraceptive methods [43]. The traditional practice of staying at home during pregnancy hinders access to healthcare [43]. Harmful gender norms and inequalities also significantly impact the prioritization of the rights of women and girls, particularly regarding their access to safe, quality, and affordable sexual and reproductive health services [45].

A coordinated three‐pronged intervention strategy addressing identified barriers: (1) Demand creation: Community health education programs targeting cultural barriers to antenatal care, addressing the current 90.3% gap in ANC4+ attendance through trained female community health workers who share cultural contexts with beneficiaries [45, 46]. (2) Supply strengthening: strategic deployment of skilled birth attendants to reduce the current 86% skilled attendance gap, prioritizing IDP settlements and rural areas with the highest maternal mortality rates. (3) System integration: Establishing referral linkages between community, primary, and secondary levels to address the identified delays in emergency obstetric care that contribute to 75% of maternal deaths [47].

## 6 Noncommunicable Diseases

Noncommunicable diseases (NCDs) are a growing public health concern in Somalia owing to a combination of factors, including lifestyle changes, limited access to healthcare, and an aging population [48]. The increasing burden of NCDs in Somalia threatens both individual well‐being and national development [48]. Several key factors exacerbate this issue, including unhealthy diets and physical inactivity, compounded by limited healthcare infrastructure and high cost of treatment [49].

Dietary habits in Somalia have shifted toward unhealthy, high‐calorie diets rich in saturated fats, sugars, and processed foods, leading to obesity, hypertension, and other cardiovascular disease (CVD) risk factors [49]. Additionally, insufficient physical activity, smoking, excessive alcohol consumption, high blood pressure, overweight and obesity, and abnormal blood lipid levels further increase the prevalence of NCDs [50]. Increased blood pressure, dyslipidemia, and smoking account for most heart attacks and strokes [50]. Studies also suggest that age, sex, and education level influence an individual's risk of developing NCDs [50, 51].

The increasing prevalence of NCDs, including cancer, threatens individual well‐being and national development [48]. Esophageal cancer is the most common cancer in Somalia, affecting male and female patients equally [52, 53]. Liver cancer is the second most common cancer, occurring more often in men [48, 53]. In addition to cancer, an increasing number of NCDs, such as hypertension and diabetes, pose a serious risk to individual health [51, 52]. A study conducted among adult internally displaced persons (IDPs) residing in camps in Baidoa found the prevalence of undiagnosed hypertension to be approximately 17% [54]. Similarly, the prevalence of diabetes in Somalia has been steadily rising, from an estimated 4.8% in 2016 to 6.5% in 2021 [48]. The contributing factors include both genetic predisposition and environmental influences [55].

Despite the growing burden of NCDs, Somalia's healthcare infrastructure is significantly constrained by limited resources, inadequate facilities, and a shortage of trained healthcare professionals [52]. This impedes timely access to quality health care services and essential medications [50]. Additionally, the high cost of NCD treatment and medication poses a substantial barrier, particularly for those living in poverty [48]. Many individuals are unable to afford necessary care, leading to untreated conditions and worsening health outcomes [55].

Addressing these challenges requires a multifaceted approach. Public health campaigns and educational initiatives play a crucial role in raising awareness of NCD risk factors and encouraging preventive measures [48, 49]. These efforts should prioritize early detection, lifestyle modifications, and community engagement to foster healthier behaviors [48]. Furthermore, implementing effective policies such as tobacco control measures and regulations on unhealthy food marketing can significantly reduce NCD risk factors [55].

Engaging communities in NCD prevention and management is vital for the success of interventions [52]. Community health education programs and outreach activities can empower individuals to take control of their own health [52]. Additionally, addressing the cultural beliefs and practices that hinder the adoption of healthy behaviors is crucial [48]. Community leaders and healthcare workers can play key roles in dispelling myths and promoting healthy practices [48]. By adopting a comprehensive and collaborative approach, Somalia could mitigate the growing burden of NCDs and improve the overall health and well‐being of its population [48, 55].

## 7 Conclusion

Somalia's public health landscape remains burdened by systemic challenges rooted in prolonged conflict, fragile infrastructure, and environmental vulnerability. Key issues, such as inadequate water, sanitation, and hygiene (WASH), rampant malnutrition, infectious disease outbreaks, high maternal and child mortality, and rising NCDs demand urgent, coordinated action. Strengthening health systems requires a resilient infrastructure, expanded access to essential services, and investment in local healthcare capacity. Addressing these challenges hinges on sustainable funding, multisectoral collaboration, and equitable policies to bridge urban‐rural and gender disparities. A unified approach, rooted in cooperation between the Somali government, international actors, and local communities, is essential to foster health equity and resilience. By harmonizing resource sharing, policy development, and grassroots empowerment, Somalia could transform its public health trajectory. This collective commitment will pave the way for a healthier and more equitable future, ensuring that public health remains a cornerstone of national recovery and development.

### 7.1 Study Limitations

This analysis acknowledged several methodological limitations that should be considered when interpreting the findings. First, it relied primarily on secondary data sources and published literature, limiting the depth of contextual analysis possible for some interventions [56]. Second, the geographic scope of available evidence shows a significant bias toward urban areas, particularly Mogadishu, potentially limiting generalizability to rural and nomadic populations that comprise 60% of Somalia's population [56]. Third, the cross‐sectional nature of most available data prevents the establishment of causal relationships between the interventions and outcomes. The security constraints in certain regions of Somalia limit comprehensive data collection, potentially underrepresenting the health challenges in areas controlled by non‐state actors. Additionally, the heavy reliance on international organizations for health service delivery creates a potential reporting bias toward interventions supported by external partners [57]. Fourth, the analysis lacks cost‐effectiveness data for most proposed interventions, limiting practical implementation guidance in resource‐constrained settings. Finally, the rapidly evolving political and security situation in Somalia means that some contextual factors may have changed since the data collection period of the cited studies.

### 7.2 Recommendations for Future Research

Building on this analysis, several research priorities have emerged that could significantly advance Somalia's public health evidence‐based and policy development. Priority areas include:

#### 7.2.1 Health Systems Research

Longitudinal studies evaluating the effectiveness of different health service delivery models (public vs. private vs. NGO‐led) in the Somali context with particular attention to sustainability and equity outcomes [56]. Implementation science research examining the successful scale‐up of community health worker programs, drawing from successful regional examples [58].

#### 7.2.2 Intervention Studies

Randomized controlled trials of culturally adapted interventions for maternal health behavior change, addressing the significant gaps in antenatal care utilization. Cost‐effectiveness analyses of the different WASH intervention models in drought‐prone areas are essential for optimal resource allocation [57, 59].

#### 7.2.3 Technology and Innovation

Studies evaluating mobile health (mHealth) interventions for disease surveillance and health service delivery in remote areas, building on Somalia's robust telecommunications infrastructure. Evaluation of drone technology for medical supply delivery to inaccessible regions during conflicts or natural disasters.

## Author Contributions

Yusuf Hared Abdi conceptualized the study. Yakub Burhan Abdullahi, Sharmake Gaiye Bashir, Mohamed Sharif Abdi, and Naima Ibrahim Ahmed prepared the first draft of this manuscript. All authors have reviewed and approved the final manuscript. All authors contributed to the manuscript review and approved the final version of the manuscript.

## Conflicts of Interest

The authors declare no conflicts of interest.

## Ethics Statement

This study did not involve any human or animal subjects and thus did not require review by an Institutional Review Board (IRB).

## Data Availability

This narrative review synthesizes previously published and publicly available sources. No new datasets were generated or analyzed. All data supporting this study are contained within the article and its referenced sources.
